# Analyzing the Carotenoid Composition of Melilot (*Melilotus officinalis* (L.) Pall.) Extracts and the Effects of Isolated (All-*E*)-lutein-5,6-epoxide on Primary Sensory Neurons and Macrophages

**DOI:** 10.3390/molecules26020503

**Published:** 2021-01-19

**Authors:** Györgyi Horváth, Eszter Csikós, Eichertné Violetta Andres, Tímea Bencsik, Anikó Takátsy, Gergely Gulyás-Fekete, Erika Turcsi, József Deli, Éva Szőke, Ágnes Kemény, Maja Payrits, Lajos Szente, Marianna Kocsis, Péter Molnár, Zsuzsanna Helyes

**Affiliations:** 1 Department of Pharmacognosy, Faculty of Pharmacy, University of Pécs, 7624 Pécs, Hungary; csikos.eszter@gytk.pte.hu (E.C.); violetta.andres@gmail.com (E.V.A.); timea.bencsik@aok.pte.hu (T.B.); Jozsef.Deli@aok.pte.hu (J.D.); peter.molnar@aok.pte.hu (P.M.); 2 Department of Biochemistry and Medical Chemistry, Medical School, University of Pécs, 7624 Pécs, Hungary; Aniko.Takatsy@aok.pte.hu (A.T.); gergely.gulyas@aok.pte.hu (G.G.-F.); Erika.Turcsi@aok.pte.hu (E.T.); 3 Department of Pharmacology and Pharmacotherapy, Medical School, University of Pécs, 7624 Pécs, Hungary; eva.szoke@aok.pte.hu (É.S.); kemeny.agnes@pte.hu (Á.K.); payrits.maja@gmail.com (M.P.); zsuzsanna.helyes@aok.pte.hu (Z.H.); 4 Department of Medical Biology and Central Electron Microscope Laboratory, Medical School, University of Pécs, 7624 Pécs, Hungary; 5 Szentágothai Research Centre, Centre for Neuroscience, University of Pécs, 7624 Pécs, Hungary; 6 Cyclolab Ltd., 1097 Budapest, Hungary; szente@cyclolab.hu; 7 Department of Plant Biology, Institute of Biology, Faculty of Sciences, University of Pécs, 7624 Pécs, Hungary; mkocsis@gamma.ttk.pte.hu

**Keywords:** *Melilotus officinalis* (L.) Pall., Fabaceae, carotenoid, (all-*E*)-lutein-5,6-epoxide-RAMEB complex, anti-inflammatory

## Abstract

*Melilotus officinalis* is known to contain several types of secondary metabolites. In contrast, the carotenoid composition of this medicinal plant has not been investigated, although it may also contribute to the biological activities of the drug, such as anti-inflammatory effects. Therefore, this study focuses on the isolation and identification of carotenoids from Meliloti herba and on the effect of isolated (all-*E*)-lutein 5,6-epoxide on primary sensory neurons and macrophages involved in nociception, as well as neurogenic and non-neurogenic inflammatory processes. The composition of the plant extracts was analyzed by high performance liquid chromatography (HPLC). The main carotenoid was isolated by column liquid chromatography (CLC) and identified by MS and NMR. The effect of water-soluble lutein 5,6-epoxide-RAMEB (randomly methylated-β-cyclodextrin) was investigated on Ca^2+^-influx in rat primary sensory neurons induced by the activation of the transient receptor potential ankyrin 1 receptor agonist to mustard-oil and on endotoxin-induced IL-1β release from isolated mouse peritoneal macrophages. (all-*E*)-Lutein 5,6-epoxide significantly decreased the percent of responsive primary sensory neurons compared to the vehicle-treated stimulated control. Furthermore, endotoxin-evoked IL-1β release from macrophages was significantly decreased by 100 µM lutein 5,6-epoxide compared to the vehicle-treated control. The water-soluble form of lutein 5,6-epoxide-RAMEB decreases the activation of primary sensory neurons and macrophages, which opens perspectives for its analgesic and anti-inflammatory applications.

## 1. Introduction

*Melilotus officinalis* (L.) Pall. (Fabaceae), an annual or biennial plant, is native to Europe and has been introduced to North America, Africa, and Australia. It possesses a sweet odor, intensified by drying, due to its coumarin content. *Meliloti* herba, the dried flowering tops of *M. officinalis*, is an official medicinal plant drug in the European and Hungarian Pharmacopoeias. The main characteristic constituents are coumarin, 3,4-dihydrocoumarin (melilotin), scopoletin and umbelliferone. Other constituents include flavonoids (mostly kaempferol and quercetin glycosides), triterpene saponins, phenolic acids (caffeic acid, melilotic acid = *o*-dihydro-coumaric acid), and essential oil [1]. According to the Community Herbal Monograph of European Medicines Agency (EMA), *M. officinalis* can be used externally and internally to relieve symptoms of discomfort and heaviness of legs related to minor venous circulatory disturbances. Furthermore, *M. officinalis* topical treatment is beneficial for bruises, sprains and insect bites (Emplastrum Meliloti) [2] and its extract Semelil (Angipars™) has positive effect in the treatment of diabetic foot ulcer [3]. As described above, flavonoids have already been identified in the yellow flowering top of *M. officinalis*, but we suggest that carotenoids may also contribute to its color and biological activities. Epidemiological studies have confirmed that carotenoids have essential protective, preventive or curative effects in case of severe diseases, e.g., different types of cancer, coronary artery diseases, or eye diseases (e.g., Age-related Macular Degeneration, AMD) [4].

We provide evidence that carotenoids, with special emphasis on lutein, exert potent inhibitory actions on both neurogenic and non-neurogenic inflammatory mechanisms by being incorporated into membranes of sensory nerves and inflammatory cells and modulating lipid rafts around certain ion channels [5,6]. The Transient Receptor Potential (TRP) receptors are nonselective cation channels expressed in a large population of polymodal nociceptors, and activated by a variety of thermal and chemical stimuli [7,8]. The TRP Ankyrin 1 (TRPA1) receptor is an important cold-gated and mechanical transduction channel co-localized with the well-known TRP Vanilloid 1 (TRPV1) pain sensing “capsaicin” receptor [9] in a subset of sensory neurons. Several exogenous and endogenous ligands can open TRPA1, such as formaldehyde, 4-hydroxynonenal, allicin, cinnamaldehyde and mustard oil (MO) [9]. TRPV1 and TRPA1 play crucial regulatory roles in pain sensation [10,11], as well as the release of pro-inflammatory neuropeptides mediating neurogenic inflammation [12,13]. Neurogenic inflammation initiated by sensory neurons plays an important role in the pathophysiological mechanisms of psoriasis, inflammatory bowel diseases, asthma, and arthritis [14]. The presently available conventional anti-inflammatory and analgesic drugs are not able to affect the neurogenic inflammatory component of these diseases, therefore, there is a great need to identify new targets. Lipid rafts are microdomains rich in cholesterol, sphingomyelin and gangliosides and modulate ion channel functions [15,16]. Our research group described that lipid rafts surrounding the TRPV1 and TRPA1 channels regulate their opening properties [17,18], and that lutein modified the lipid raft functions [5].

Carotenoids are non-water-soluble compounds, therefore their investigation in different assays is difficult. Their water-solubility can be increased with cyclodextrins, e.g., RAMEB (randomly methylated β-cyclodextrin (BCD)). RAMEB has about 13–14 methoxy groups per BCD molecule. The distribution of methyl groups on the BCD-ring is statistically random on both the primary and secondary face of the macrocycle. Methylation of BCD results in an amorphous solid compound, which has improved water solubility and complexation efficacy of lipophilic compounds in aqueous solution [19]. The pharmaceutical application of this BCD methyl ether is mainly in the solubilization and delivery of lipophilic drug actives. It is probably the most potent solubility enhancing cyclodextrin derivative [20]. There are two approved human pharmaceutical products which have RAMEB as solubilizing excipient. There is a nasal estradiol product by Servier called Aerodiol™ based on RAMEB as solubility and absorption enhancing excipient [21]. Another RAMEB-based human pharmaceutical product is an eye drop called Clorocil™, a chloramphenicol containing product by Oftalder Co. in Poland [22].

There are data about the flavonoid, but not the carotenoid composition of *M. officinalis*, used worldwide for inflammatory and painful conditions, therefore, we first analyzed this aspect. Furthermore, the effects of the main carotenoid component, lutein 5,6-epoxide, were investigated on the activation of isolated primary sensory neurons and macrophages involved in nociceptive and inflammatory mechanisms.

## 2. Results

### 2.1. Carotenoid Composition of Meliloti Herba

**Figure 1:**
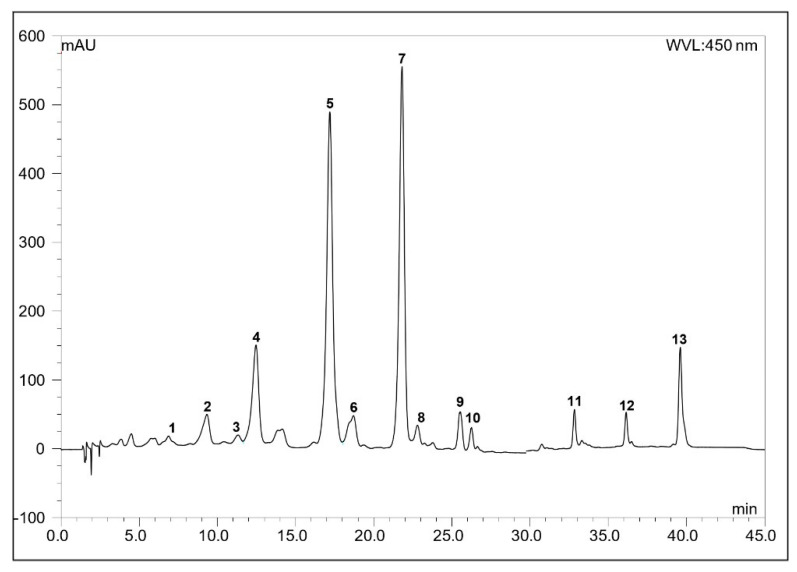
The HPLC chromatogram of the total extract of *Meliloti* herba. Detection at 450 nm; other conditions in the Material and Methods section. For peak numbers see Table 1.

The total carotenoid content was 0.26 mg/g for dry plant material and 0.035 mg/g for wet plant material. The carotenoid composition of the total extract was determined on the basis of the spectrum of the peaks, as well as the retention times (t_R_) and co-chromatography with authentic reference samples [23,24,25,26,27,28]. The HPLC chromatogram of the saponified total extract (Figure 1) showed that (all-*E*)-violaxanthin (10.5%) (peak 4), (all-*E*)-lutein 5,6-epoxide (33.8%) (peak 5), (all-*E*)- lutein (32.5%) (peak 7) and β-carotene (6.0%) (peak 13) were the most characteristic carotenoids. The detailed composition with the spectroscopic data (nm) of the total extract is shown in Table 1.

**Figure 2:**
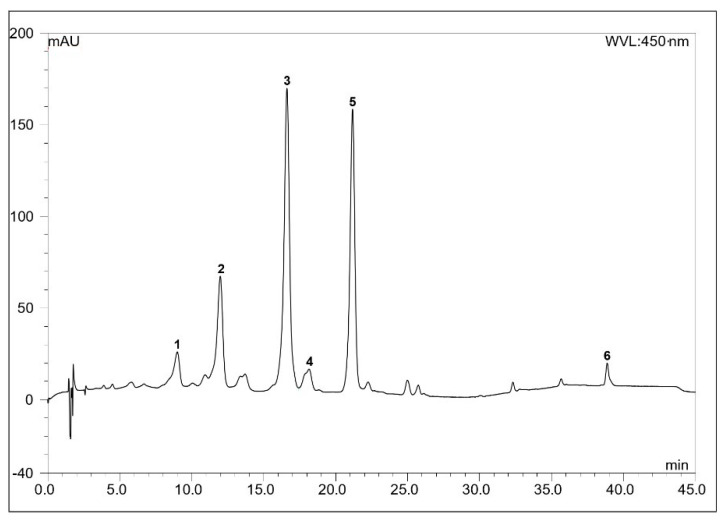
The HPLC chromatogram of the crystallized extract of *Meliloti* herba. Detection at 450 nm; other conditions in the Material and Methods section. For peak numbers see Table 2.

The total extract was crystallized with a mixture of toluene and hexane (1:5) [24,26], and the gained crystal was also analyzed by HPLC. Figure 2 and Table 2 present the carotenoid composition of the crystallized extracts: (all-*E*)-neoxanthin (3.4%) (peak 1), (all-*E*)-violaxanthin (10.5%) (peak 2), (all-*E*)-lutein 5,6-epoxide (33.8%) (peak 3), flavoxanthin + chrysanthemaxanthin (3.8%) (peak 4), (all-*E*)-lutein (32.5%) (peak 5) and β-carotene (6.0%) (peak 6).

**Figure 3:**
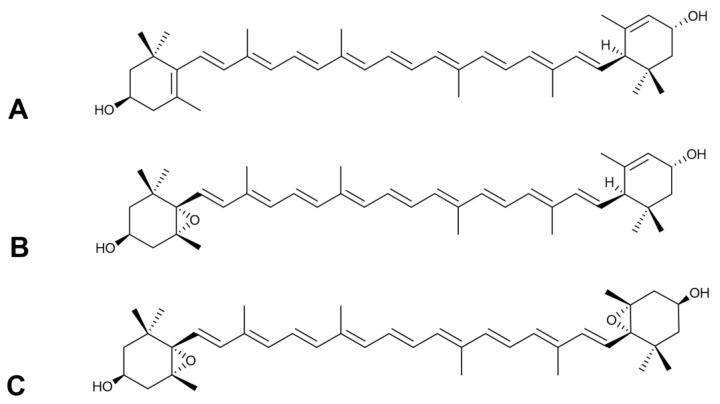
The most characteristic carotenoids of *Meliloti* herba. (**A**) (all-*E*)-lutein. (**B**) (all-*E*)-lutein 5,6-epoxide. (**C**) (all-*E*)-violaxanthin.

From the crystals, the main carotenoids could be isolated by column liquid chromatography (CLC) using a mixture of toluene-hexane (3:2 *v*/*v*) [26]. The fractions of carotenoids appeared in order of their decreasing absorption affinity on the CaCO_3_ column. The following carotenoids were isolated in crystalline state: (all-*E*)-lutein (Figure 3A) (4 mg; 97% HPLC purity; λ_max_: 420, 444, 473 nm), (all-*E*)-lutein 5,6-epoxide (Figure 3B) (6 mg; 98% HPLC purity; λ_max_: 427, 440, 471 nm), and (all-*E*)-violaxanthin (Figure 3C) (2 mg; 95% HPLC purity; λ_max_: 418, 442, 471 nm). The structures of isolated compounds were confirmed by MS and NMR spectroscopic techniques [29,30]. In all samples the protonated mass (M + H^+^), the sodium adduct (M + Na^+^) and the potassium adduct (M + K^+^) were identified (see Figures S1–S3). The results were reproducible for the sample (three replicates) and from different extractions as well. The complete 1H-NMR assignments are also given for the isolated compounds (see Figures S4–S6). The 1H-NMR data (δ(H) and *J*(H,H) values) were in accordance with the literature data [31,32].

Carotenoids are water-insoluble, therefore the main carotenoid of *Meliloti* herba, (all-*E*)-lutein 5,6-epoxide (Figure 3B), was packed into RAMEB (randomly methylated-β-cyclodextrin) to enhance its water-solubility in the in vitro functional experiments.

### 2.2. RAMEB-Lutein 5,6-Epoxide Decreases Mustard Oil-Evoked Ca^2+^-Influx in Cultured Primary Sensory Neurons

**Figure 4:**
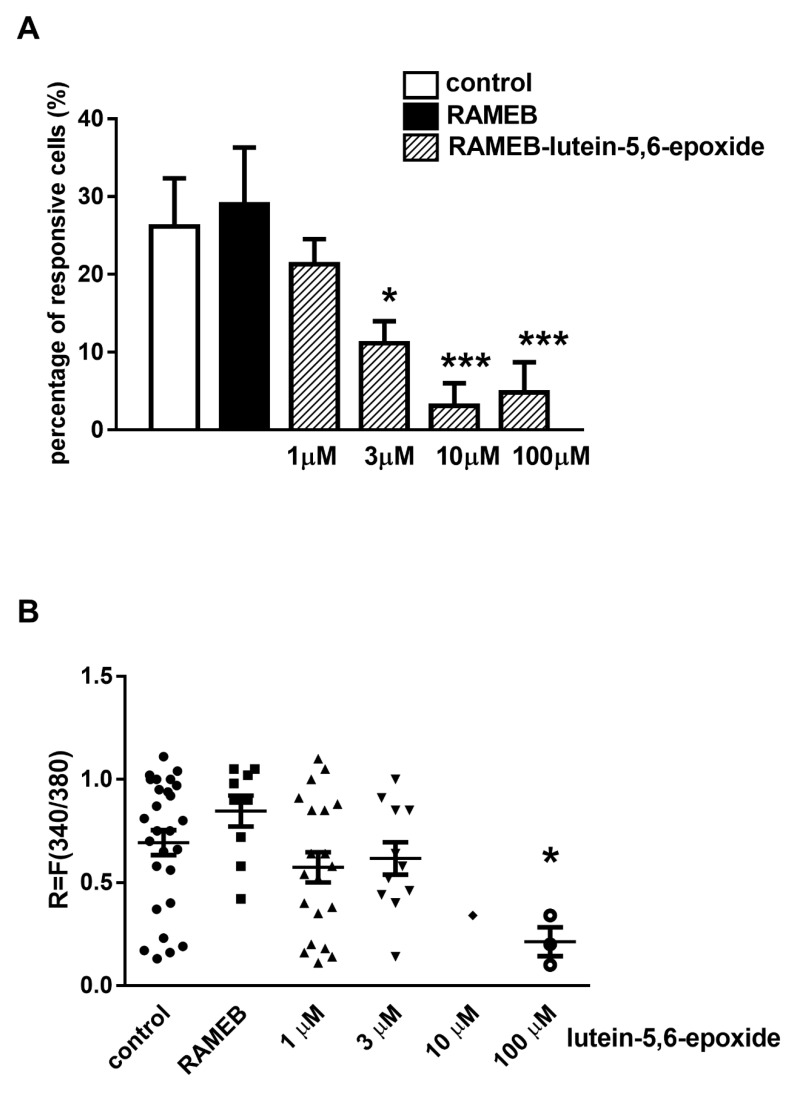
Effect of randomly methylated-β-cyclodextrin-(RAMEB)-lutein 5,6-epoxide on TRP Ankyrin 1 (TRPA1) activation-induced Ca^2+^-influx in cultured trigeminal ganglion neurons. (**A**) Columns show the percentage of neurons responding to the TRPA1 activating agent mustard oil (MO, 200 μM) in untreated control, RAMEB-solvent-treated control and 1, 3, 10 and 100 μM RAMEB-lutein 5,6-epoxide-treated cell cultures. N = 31–107 cells per group. *** *p* < 0.001, (vs. RAMEB alone, One-way ANOVA, Bonferroni’s post hoc test). (**B)** Change in the fluorescence ratio (R = F340/F380) is presented after 1–100 µM RAMEB-lutein 5,6-epoxide treatment. Dot plot represents mean + SEM * *p* < 0.05, (Kruskall-Wallis test with Dunn’s post-test, RAMEB-lutein 5,6-epoxide vs. RAMEB control). N = 31–107 cells per group.

The percentage of neurons responding with sustained Ca^2+^-influx after a 20–30 s latency to 200 µM MO activating the TRPA1 ion channel was determined as control. The ratio of mustard oil-responsive cells was 26.1% ± 6.2% (28 out of 107). Neither the proportion of MO-responsive neurons nor the fluorescence increment changed after treatment with RAMEB solvent alone in the highest concentration used for the RAMEB-lutein 5,6-epoxide solution; the respective values were 29.03% ± 7.3% (9 out of 31) and R = 0.84 ± 0.21 (Figure 4A,B). The effect of 1, 3, 10 and 100 μM RAMEB-lutein 5,6-epoxide treatment was measured on the ratio of sensory neurons responding to MO. No effect was detected after 1 μM lutein 5,6-epoxide treatment, but significant inhibition was observed after 3, 10 µM and 100 µM lutein 5,6-epoxide incubation resulting in 11.14% ± 2.85% (11 out of 102), 3.1% ± 2.9% (1 out of 32) and 4.8% ± 3.9% (3 out of 63) responsive cells, respectively (Figure 4A). The peak fluorescence increment (R) to MO was 0.69 ± 0.3, which was significantly decreased by 100 μM RAMEB-lutein 5,6-epoxide to 0.21 ± 0.09 (Figure 4B).

### 2.3. RAMEB-Lutein 5,6-Epoxide Reduces LPS-Induced IL-1β Production of Isolated Peritoneal Macrophages

**Figure 5:**
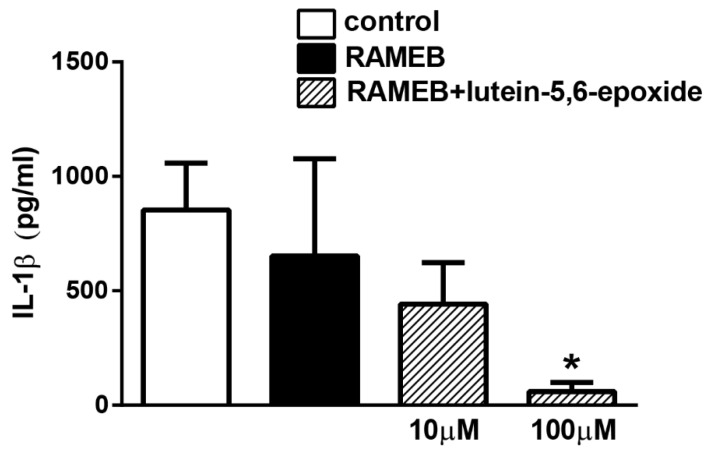
Activity of RAMEB-lutein 5,6-epoxide on lipopolysaccharide-(LPS)-induced IL-1β production. Concentration of IL-1β inflammatory cytokine in peritoneal macrophage cell culture supernatant after LPS stimulation was significantly decreased upon higher dose (100 µm) RAMEB+lutein-5,6-epoxid treatment. N = 3. * *p* < 0.05, (vs. LPS treated cells, One-way ANOVA, Tukey’s post hoc test).

LPS (lipopolysaccharide) stimulation induced 853.2 pg/mL IL-1β inflammatory cytokine release from isolated peritoneal macrophages measured in the supernatant. RAMEB treatment alone did not have any effect. RAMEB-lutein 5,6-epoxide concentration-dependently decreased IL-1β production, which was significant in the 100 µM concentration (Figure 5).

## 3. Discussion

We provide here the first description of the carotenoid composition of *M. officinalis.* By column chromatographic technique, (all-*E*)-violaxanthin, (all-*E*)-lutein 5,6-epoxide and (all-*E*)-lutein) were isolated in crystalline form, and characterized by modern spectroscopic methods (MS, NMR). Furthermore, functional evidence was demonstrated for the inhibitory effects of the main carotenoid component, (all-*E*)-lutein 5,6-epoxide, on the activation of primary sensory neurons and isolated macrophages.

We previously showed that greater celandine (*Chelidonium majus* L.) is one of the most suitable natural sources of (all-*E*)-lutein 5,6-epoxide [33]. The naturally occurring carotenoid 5,6-epoxides (e.g., lutein 5,6-epoxide, antheraxanthin, violaxanthin) play important role in the xanthophyll cycle of photosynthesis [34]. Lutein and zeaxanthin are protective in eye diseases because of absorbing damaging blue light that enters the eye [4]. Carotenoids are thought to provide health benefits by decreasing the risk of certain cancers [4,35], cardiovascular [4,36] and eye diseases [4,37,38]. These chronic disorders are all associated with inflammatory processes. The beneficial effects of carotenoids are thought to be due to their antioxidant and anti-inflammatory activities [39], but the precise mechanism of action is unclear.

The model systems we used for functional investigation of water-soluble cyclo-dextrine complex of (all-*E*)-lutein 5,6-epoxide refer to mechanisms involved in a broad range of nociceptive and inflammatory processes: TPRA1 activation is an important pathway of several inflammatory mediators and irritants on sensory neurons [12,13], and LPS-evoked Toll-like receptor 4 stimulation on macrophages mimics an important component of the inflammatory cascade [40].

(all-*E*)-Lutein 5,6-epoxide decreased the MO-induced TRPA1 receptor activation on primary sensory neurons. These results supported our previously published data showing similar inhibitory action of RAMEB-lutein complex on trigeminal ganglion (TRG) neurons and MO-induced neurogenic mouse ear swelling in vivo [5]. These data suggested that disrupting lipid rafts by depleting their various constituents, decreased the MO-induced opening properties of the TRPA1 cation channel [18]. This concept is supported by the fact that polar carotenoids containing hydroxylated α- and β-ionon rings can be perfectly incorporated into membrane bilayers due to their rod-like structure, polar end groups and the molecular dimensions matching the thickness of the bilayer [41]. RAMEB-lutein 5,6-epoxide might also be able to influence the hydrophobic interactions between the TRPA1 ion channel and membrane lipid raft constituents, and therefore to modulate the gating of TRPA1.

Previous studies showed that coumarin and a standardized extract from the flowers of *M. officinalis* containing 0.25% coumarin showed anti-inflammatory activities by reducing the activation of circulating phagocytes and citrulline production [42,43]. Therefore, we propose that the main characteristic carotenoid of *M. officinalis*, (all-*E*)-lutein 5,6-epoxide, could also contribute to its anti-inflammatory effect. In one study, ethanol and ethyl acetate extracts including carotenoids of *Scutellaria barbata* could significantly inhibit the production of LPS-induced nitric oxide, prostaglandin E2, IL-6, and IL-1β, as well as the expressions of phosphor extracellular signal-regulated kinase and phosphor-c-Jun N-terminal kinase (p-JNK), but failed to influence tumor necrosis factor-α expression. Both extracts had a concentration-dependent anti-inflammatory activity on macrophage cell line (RAW 264.7) [44]. Our results support that lutein 5,6-epoxide reduces LPS-induced IL-1β production.

Further studies are planned to explore the pharmacological effects and the mode of action of this carotenoid under in vivo conditions to support and broaden its preventive and therapeutic use in the clinical practice.

## 4. Materials and Methods

### 4.1. Plant Material and Extraction of Carotenoids

The flowering tops of *M. officinalis* were collected near Pellérd (southwest part of Hungary) in July 2013. The voucher specimen (Mo01) was deposited at the Herbarium of the Department of Pharmacognosy, University of Pécs (Pécs, Hungary). The authentication of the plant material was done according to the requirements of the European Pharmacopoeia 6.0.

The isolation of carotenoids was performed in darkened laboratory and at room temperature (21 °C) using methanol for dehydration of plant material, to avoid pigment decomposition [24]. The fresh flowering tops (260 g) were extracted three times with methanol and once with diethyl ether. The methanol extracts were combined and transferred into the mixture of hexane and toluene (1:1) in a separating funnel. After evaporation of the latter solution the residue was dissolved in diethyl ether. The ethereal solutions were combined and this total extract was saponified in heterogeneous phase (30% potassium hydroxide in methanol) overnight. Then the plant extract was crystallized with a mixture of toluene and hexane (1:5). The composition of the total and crystallized extracts was analyzed by high performance liquid chromatography (HPLC). The main carotenoids were isolated by column liquid chromatography (CLC) [26].

### 4.2. Chemicals

All chemicals used in the extraction, in CLC and during HPLC, MS and NMR were analytical grade quality (Sigma-Aldrich Ltd., Budapest, Hungary, Scharlab Magyarország Ltd., Debrecen, Hungary). Acetonitrile (ACN), trifluoroacetic acid (TFA, ≥99%), α-cyano-4-hydroxycinnamic acid (CHCA, ≥99%) and 2,5-dihydroxybenzoic acid (DHB, ≥99%), were purchased from Sigma-Aldrich (Budapest, Hungary). Peptide calibration standard (consisting of bradykinin, angiotensin II, angiotensin I, substance P, renin substrate, ACTH clip (1–17), ACTH clip (18–39) and somatostatin) was obtained from Bruker Daltonics (Bremen, Germany). Bi-distilled water was prepared in our laboratory. In the in vitro pharmacological experiments too, chemicals with analytical grade quality were used (Sigma-Aldrich Ltd., Budapest, Hungary). For the functional studies in the cell culture systems, water soluble randomly methylated-β-cyclodextrin (RAMEB)-lutein 5,6-epoxide complex containing 1% carotenoid was prepared by Cyclolab Ltd. (Budapest, Hungary) [45].

### 4.3. Instrumentals

UV-Vis spectra were acquired with a Jasco V-530 spectrophotometer (Jasco Corporation, Tokyo, Japan). The HPLC analyses were performed with Dionex P680 quaternary analytical pump, a Dionex PDA 100 UV/vis detector (Thermo Fisher Scientific, Inc., Waltham, MA, USA) with Chromeleon 6.8 software (Thermo Fisher Scientific, Inc., Waltham, MA, USA) and a column temperature control module.

An Autoflex II MALDI instrument from Bruker Daltonics (Bremen, Germany) was employed for the mass spectrometric measurements. ^1^H-NMR spectra were recorded with a Bruker Avance III 500 (500.15 MHz for ^1^H, Bruker BioSpin GmbH, Rheinstetten, Germany) spectrometer. Chemical shifts were referenced to the residual solvent signals. Measurements were run at a probe temperature of 298 K in CDCl_3_ solutions.

### 4.4. Experimental Conditions of HPLC Analysis

Chromatograms were developed on a 250 × 4.6 mm stainless steel LiChrospher 100 RP 18e, 5 µm (Merck KGaA, Darmstadt, Germany) column, with 1.25 mL/min flow rate, at 22 °C. Eluents were (A) H_2_O/MeOH = 12/88 *v*/*v*%, (B) MeOH and (C) Acetone/MeOH = 50/50 *v*/*v*%. The gradient program was the following: 0–2 min 100% A, 2–10 min to 80% A/20%B, 10–18 min to 50% A/50% B,18–25 min to 100% B, 25–27 min 100% B, 27–33 min to 100% C, 33–38 min 100% C, 38–40 min to 100% B (in linear steps). The peak area was used to determine the percentage of individual compounds in the extracts [28].

### 4.5. Experimental Conditions of CLC Separation

In CLC separation, glass columns with length of 30 cm and inner diameter of 6 cm were used. The adsorbent CaCO_3_ was pharmacopeial quality (Ph. Hg. VI., Biogal, Debrecen, Hungary). Toluene-hexane mixture (3:2 *v*/*v*) was used as eluent [23,26].

### 4.6. Identification of Carotenoids

The total carotenoid content of *Meliloti* herba was determined by UV-Vis spectrophotometry [24]. The individual carotenoids were identified on the basis of their UV-Vis spectroscopic properties in different solvents, chemical reactions [(*E*/*Z*)-isomerization, 5,6-epoxide → 5,8-epoxide (furanoid oxide) rearrangement] [23,24,25,26,27], by co-chromatography with authentic reference samples [24] and by HPLC retention times. The authentic reference samples were taken from the collection of the traditional Carotenoid Research Group of the Department of Biochemistry and Medical Chemistry, University of Pécs (Hungary). The identification methods mentioned in this paragraph fulfill the minimum identification criteria described in [24]. Moreover, the isolated carotenoids of *Meliloti* herba were also identified by MS [29] and NMR [30].

### 4.7. Mass Spectrometry

#### 4.7.1. Preparation of Different Matrices

Two different matrices were used for the ionisation of the samples of isolated carotenoids. DHB was prepared in 50 *v*/*v*% acetonitrile and 0.1% TFA in water at a concentration of 25 mg/mL and 10 mg CHCA was dissolved in 300 µL of 50% ACN and 0.1% TFA in water.

#### 4.7.2. MALDI-TOF/MS (Matrix-Assisted Laser Desorption/Ionization-Time of Flight Mass Spectrometry) Conditions and Measurements

All mass spectra were monitored in positive mode with pulsed ionisation (λ = 337 nm; nitrogen laser, maximum pulse rate: 50 Hz; maximal intensity 20–30% of the laser for peptides). Samples were measured in reflectron mode using a delayed extraction of 120 ns. The accelerating voltage was set to +19 kV, and the reflectron voltage was set to +20 kV. Spectra were the sum of 1000 shots, and external calibration has been implemented. Data processing was executed with Flex Analysis software packages (version: 2.4., Bruker BioSpin GmbH, Rheinstetten, Germany).

External calibration was used before every measurement. The instrument was calibrated for the typical masses of the matrixes and for peptide calibration standard mixture. The samples were dissolved in ACN. Dried droplet method was used; 1 µL of sample was put on a steel target plate and 1 µL matrix was mixed with it. The mixture was dried at room temperature and then MS analysis was completed.

### 4.8. In Vitro Investigation of the Effects of Isolated Lutein 5,6-Epoxide

#### 4.8.1. Ethics Statement

To investigate the effect of lutein 5,6-epoxide in vitro, cultures were prepared from animals. Animals were bred and kept in the Laboratory Animal House of the Department of Pharmacology and Pharmacotherapy, the University of Pécs. The optimal parameters were provided for the animals (e.g., 325 × 170 × 140 mm cages, 12 h light/dark cycle, 24–25 °C, chow, water).

This study was carried out in strict accordance with the following recommendations: European legislation (Directive 2010/63/EU) and Hungarian Government regulation (40/2013., II. 14.) on the protection of animals used for scientific purposes and complied with the recommendations of the International Association for the Study of Pain. The studies were approved by the Ethics Committee on Animal Research of University of Pécs (license No.: BA02/2000-26/2018). Cultures made from 1–3 days old Wistar rat pups were also approved by this Committee. All surgery of mice was performed under deep ketamine (100 mg/kg i.p.) and xylazine (5 mg/kg i.p.) anaesthesia and animals were exsanguinated by decapitation. All efforts were made to minimize suffering.

#### 4.8.2. Primary Cultures of Trigeminal Ganglion (TRG) Neurons

Cultures were made from 1–3 days old Wistar rat pups. Trigeminal ganglia were dissected in phosphate-buffered solution (PBS), incubated for 35 min at 37 °C in collagenase (Type XI, 1 mg/mL) and then in deoxyribonuclease I (1000 units/mL) for 8 min. The ganglia were then rinsed with Ca^2+^ and Mg^2+^ free PBS and dissociated by trituration. TRG cells were plated on poly-d-lysin-coated glass coverslips and grown in a nutrient-supplemented medium. The coverslips were maintained at 37 °C in a humid atmosphere with 5% CO_2_. Nerve growth factor (NGF, 200 ng/mL) was added, as described earlier [17].

#### 4.8.3. Ratio-Metric Technique of Intracellular Free Calcium Concentration [Ca^2+^]_I_ Measurement with the Fluorescent Indicator Fura-2 AM

Two-three-day-old cell cultures were stained for 30 min at 37 °C with 1 µM fluorescent Ca^2+^ indicator dye, fura-2-AM (Molecular Probes, Eugene, OR, USA). Dye loading was followed by at least 5 min wash in extracellular solution (ECS) at room temperature. Calcium transients of TRG neurons to MO were examined with a fluorescence microscope (Olympus BX50WI, Tokyo, Japan) as described elsewhere [17]. Fluorescence images were taken with an Olympus LUMPLAN FI/x20 0.5 W water immersion objective and a digital camera (CCD, SensiCam PCO, Kelheim, Germany), connected to a computer. Cells were illuminated alternately at 340 and 380 nm light (for 50 to 400 ms each) generated by a monochromator (Polychrome II., Till Photonics, Kaufbeuren, Germany) under the control of the Axon Imaging Workbench (AIW, Los Altos, CA, USA). Emitted light >510 nm was measured. The R = F340/F380 was monitored (rate 1Hz) continuously for up to 5 min, while a few sample images were also recorded. The R values generated by the Axon Imaging Workbench 2.1 (AIW, Axon Instruments, Los Altos, CA, USA) software were then processed by the Origin software version 7.0 (Originlab Corp., Northampton, MA, USA). Baseline fluorescence was monitored for 20 s at least before drug administration. MO (Sigma, St. Louis, MO, USA) was used in 200 μM. Neurons were incubated with different concentrations of RAMEB-lutein 5,6-epoxide (1, 3, 10 and 100 µM) or the respective RAMEB-vehicle for 60 min, at 37 °C in a humid atmosphere with 5% CO_2_, or were untreated controls.

#### 4.8.4. Primary Cultures of Peritoneal Macrophages and Measurement of Interleukin-1β (IL-1β)

Peritoneal macrophages were obtained from the peritoneal cavity of 8-week-old male NMRI mice 4 h after i.p. endotoxin (lipopolysaccharide: LPS) injection (300 µL of 300 µg/mL solution per animal, *Salmonella enterica* LPS, Sigma-Aldrich, St. Louis, MO, USA). Four hours later mice were exsanguinated under deep ketamine and xylazine anaesthesia. The abdominal cavity was leached using 3 mL cell culture medium (RPMI 1640, Sigma, St. Louis, MO, USA) supplemented with 10% foetal bovine serum under sterile conditions. The lavage fluid was collected into ice-cold tubes and cell count was determined in the samples with a haemocytometer. In the next step 100 μL lavage fluid samples, 1 µL LPS solution and 100 µL of test compounds were added into 800 μL culture medium in a 24-well plate. The plates were then incubated in CO_2_-incubator at 37 °C. Well contents were collected 8 h later and centrifuged for 5 min at 12,500 rpm. The concentration of the inflammatory cytokine IL-1β was measured from the supernatants by sandwich ELISA method using IL-1β BD OptEIA ELISA set (cat. Nr. 559603, BD Biosciences Eastern Europe, Heidelberg, Germany) [46].

### 4.9. Statistical Analysis

Data reported in this paper are the means ± SEM of at least three independent experiments. Statistical analysis was performed by one-way ANOVA followed by Bonferroni’s post hoc test in cases of the responsive neuronal percentage activation and the IL-1β-release from the macrophages showing normal distribution. The non-parametric Kruskal-Wallis test with the Dunn’s post hoc test was used for analysis of R values. In all cases *p* ≤ 0.05 was considered statistically significant.

## 5. Conclusions

The water-soluble form of the main carotenoid component of *Melilotus officinalis*, lutein 5,6-epoxide-RAMEB, decreases the activation of primary sensory neurons and macrophages, which opens perspectives for its analgesic and anti-inflammatory applications.

## Figures and Tables

**Table 1:** The carotenoid composition of the total extract of *Meliloti* herba.

Carotenoid	Peak Number	t_R_ (min)	%	UV-Vis λ_max_ (nm) in High Performance Liquid Chromatography (HPLC) Solvent		
Unidentifiable mixture	1	6.9	1.0	401	424	444
(all-*E*)-neoxanthin	2	9.4	3.4	417	440	469
(9*Z*)-neoxanthin	3	11.3	0.8	413	436	465
(all-*E*)-violaxanthin	4	12.5	10.5	416	438	468
(all-*E*)-lutein 5,6-epoxide	5	17.2	33.8	415	438	468
flavoxanthin + chrysanthemaxanthin	6	18.7	3.8	398	420	447
(all-*E*)-lutein	7	21.8	32.5	(418)	443	471
(13*Z*) + (13′*Z*)-lutein 5,6-epoxide	8	22.8	1.5	409	432	460
(9*Z*) + (9′*Z*)-lutein	9	25.5	2.6	(414)	439	466
(13*Z*) + (13′*Z*)-lutein	10	26.3	1.2	(412)	437	463
α-cryptoxanthin	11	32.8	1.6	(420)	445	473
(9*Z*) + (9′*Z*)-β-cryptoxanthin	12	36.2	1.3	(420)	447	473
β-carotene	13	39.6	6.0	(423)	452	478

**Table 2:** The carotenoid composition of the crystallized extract of *Meliloti* herba.

Carotenoid	Peak Number	t_R_ (min)	%	UV-Vis λ_max_ (nm) in HPLC Solvent		
(all-*E*)-neoxanthin	1	9.0	3.4	417	441	469
(all-*E*)-violaxanthin	2	12.0	10.5	416	439	468
(all-*E*)-lutein 5,6-epoxide	3	16.6	33.8	415	438	467
flavoxanthin + chrysanthemaxanthin	4	18.2	3.8	398	421	447
(all-*E*)-lutein	5	21.2	32.5	(418)	443	470
β-carotene	6	38.9	6.0	(423)	452	478

## Data Availability

The data presented in this study are available in this article and its supplementary material.

## Supplementary Materials

The following are available online. Figure S1: Mass spectrum of (all-*E*)-lutein, Figure S2: Mass spectrum of (all-*E*)-lutein-5,6-epoxide, Figure S3: Mass spectrum of (all-*E*)-violaxanthin, Figure S4: ^1^H-NMR spectrum of the isolated (all-*E*)-lutein, Figure S5: ^1^H-NMR spectrum of the isolated (all-*E*)-lutein 5,6-epoxide, Figure S6: ^1^H-NMR spectrum of the isolated (all-*E*)-violaxanthin.
