# Supplementary material for: Analyzing the Carotenoid Composition of Melilot (Melilotus officinalis (L.) Pall.) Extracts and the Effects of Isolated (All-E)-lutein-5,6-epoxide on Primary Sensory Neurons and Macrophages
Source: Molecules. 2021 Jan 19;26(2):503. doi: 10.3390/molecules26020503 (PMC7832904; doi:10.3390/molecules26020503)
Supplement: Supplementary file 1 [file molecules-26-00503-s001.pdf]

Supplementary Material

Analysing the carotenoid composition of Melilot (*Melilotus officinalis* (L.) Pall.) extracts and the effects of isolated (all-*E*)-lutein-5,6-epoxide on primary sensory neurons and macrophages

Györgyi Horváth[1,*], Eszter Csikós[1], Eichertné Violetta Andres[1], Tímea Bencsik[1], Anikó Takátsy[2], Gergely Gulyás-Fekete[2], Erika Turcsi[2], József Deli[1,2], Éva Szőke[3], Ágnes Kemény[3,4,5], Maja Payrits[3,5], Lajos Szente[6], Marianna Kocsis[7], Péter Molnár[1], Zsuzsanna Helyes[3,5]

1 Department of Pharmacognosy, Faculty of Pharmacy, University of Pécs, Pécs, Hungary
2 Department of Biochemistry and Medical Chemistry, Medical School, University of Pécs, Pécs, Hungary
3 Department of Pharmacology and Pharmacotherapy, Medical School, University of Pécs, Pécs, Hungary
4 Department of Medical Biology and Central Electron Microscope Laboratory, Medical School, University of Pécs, Pécs, Hungary
5 Szentágothai Research Centre, Centre for Neuroscience, University of Pécs, Pécs, Hungary
6 Cyclolab Ltd., Budapest, Hungary
7 Department of Plant Biology, Institute of Biology, Faculty of Sciences, University of Pécs, Pécs, Hungary
* Correspondence: horvath.gyorgyi@gytk.pte.hu; Tel.: +36-72-503650-28823

Figure S1. Mass spectrum of (all-*E*)-lutein

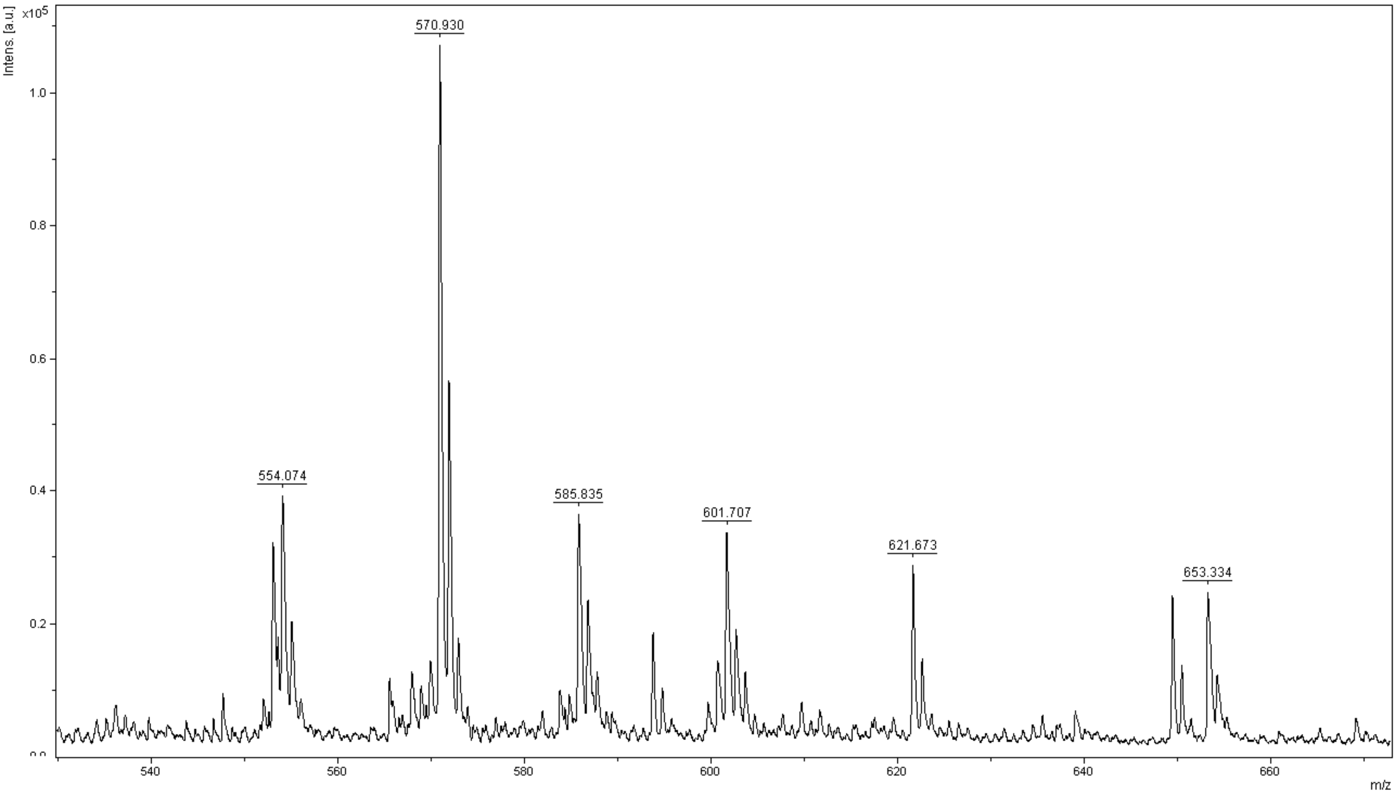

Figure S2. Mass spectrum of (all-*E*)-lutein-5,6-epoxide

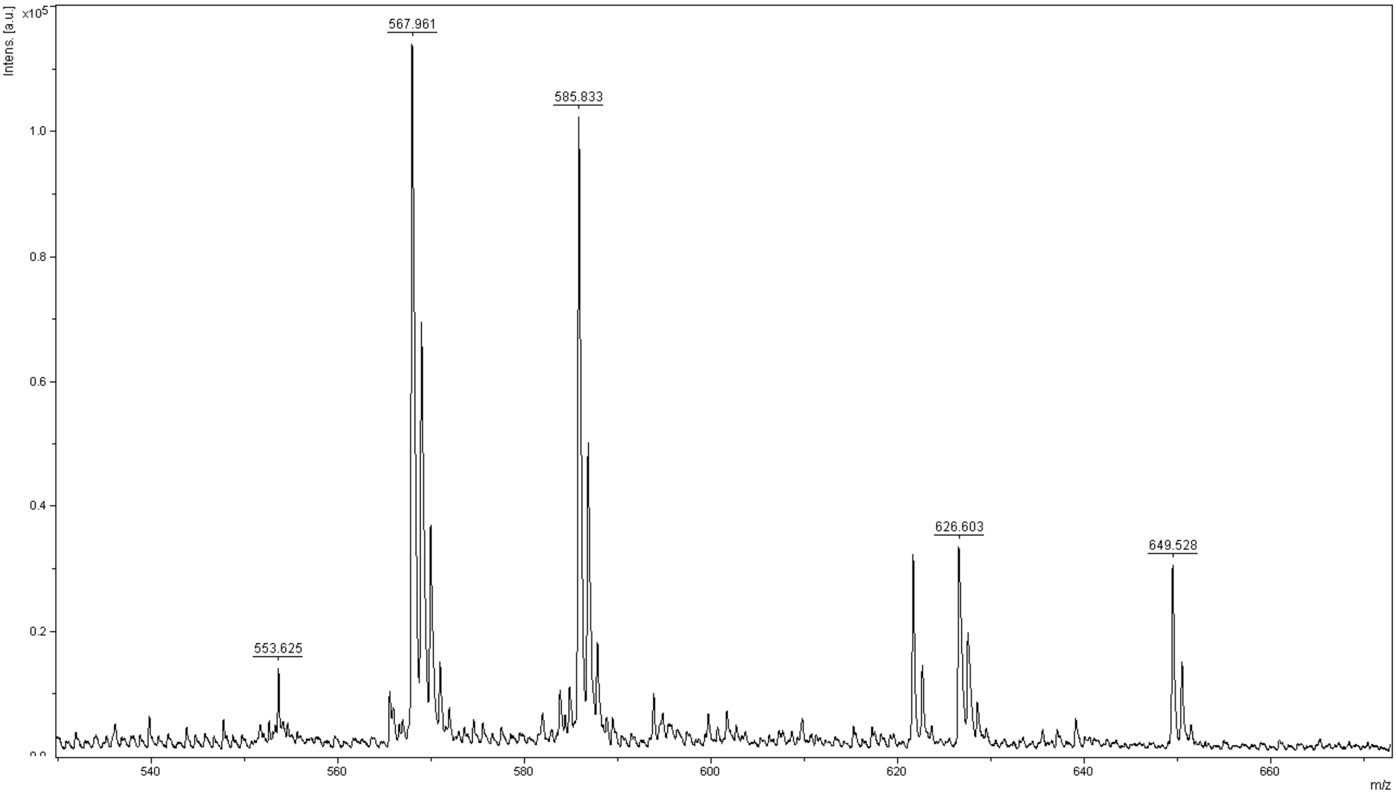

Figure S3. Mass spectrum of (all-*E*)-violaxanthin

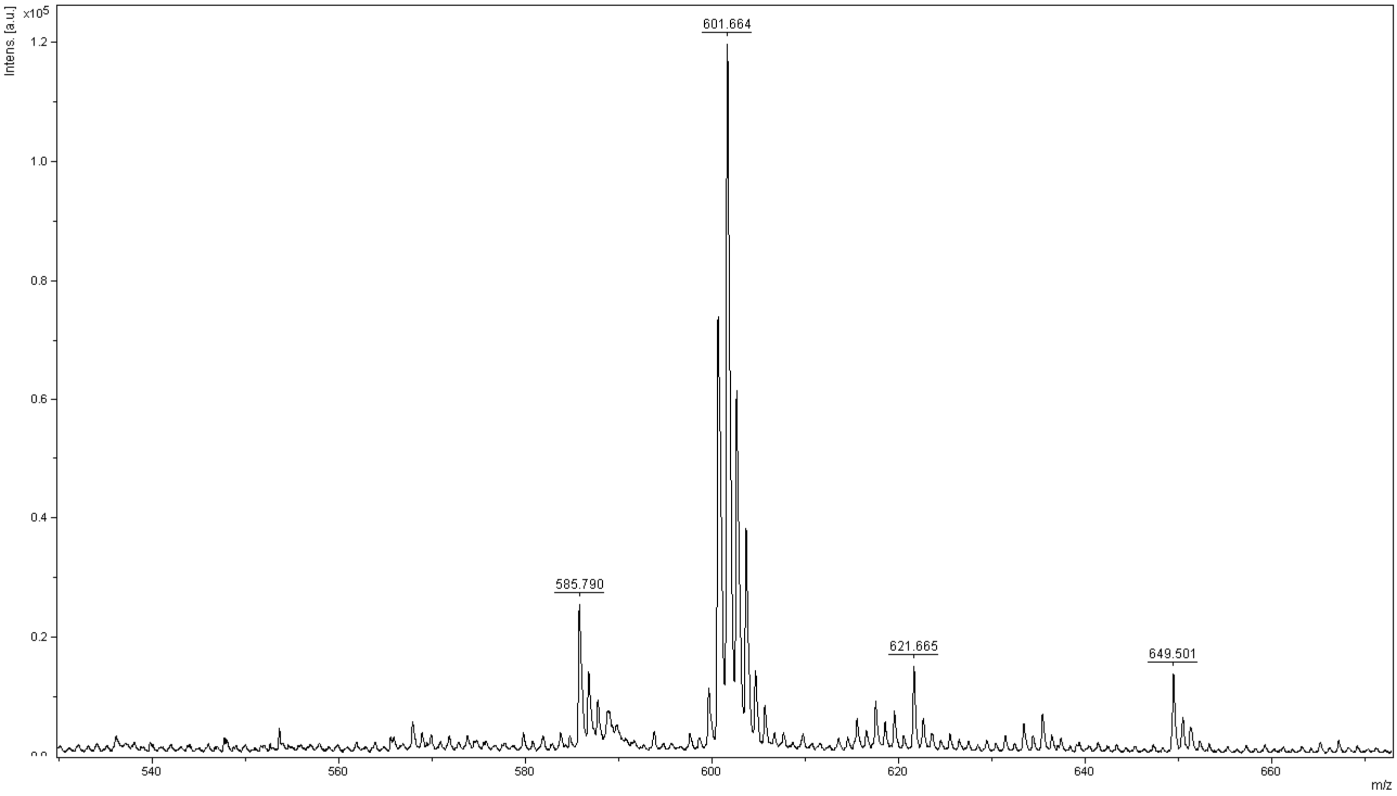

Figure S4. ^{1}H-NMR spectrum of the isolated (all-*E*)-lutein

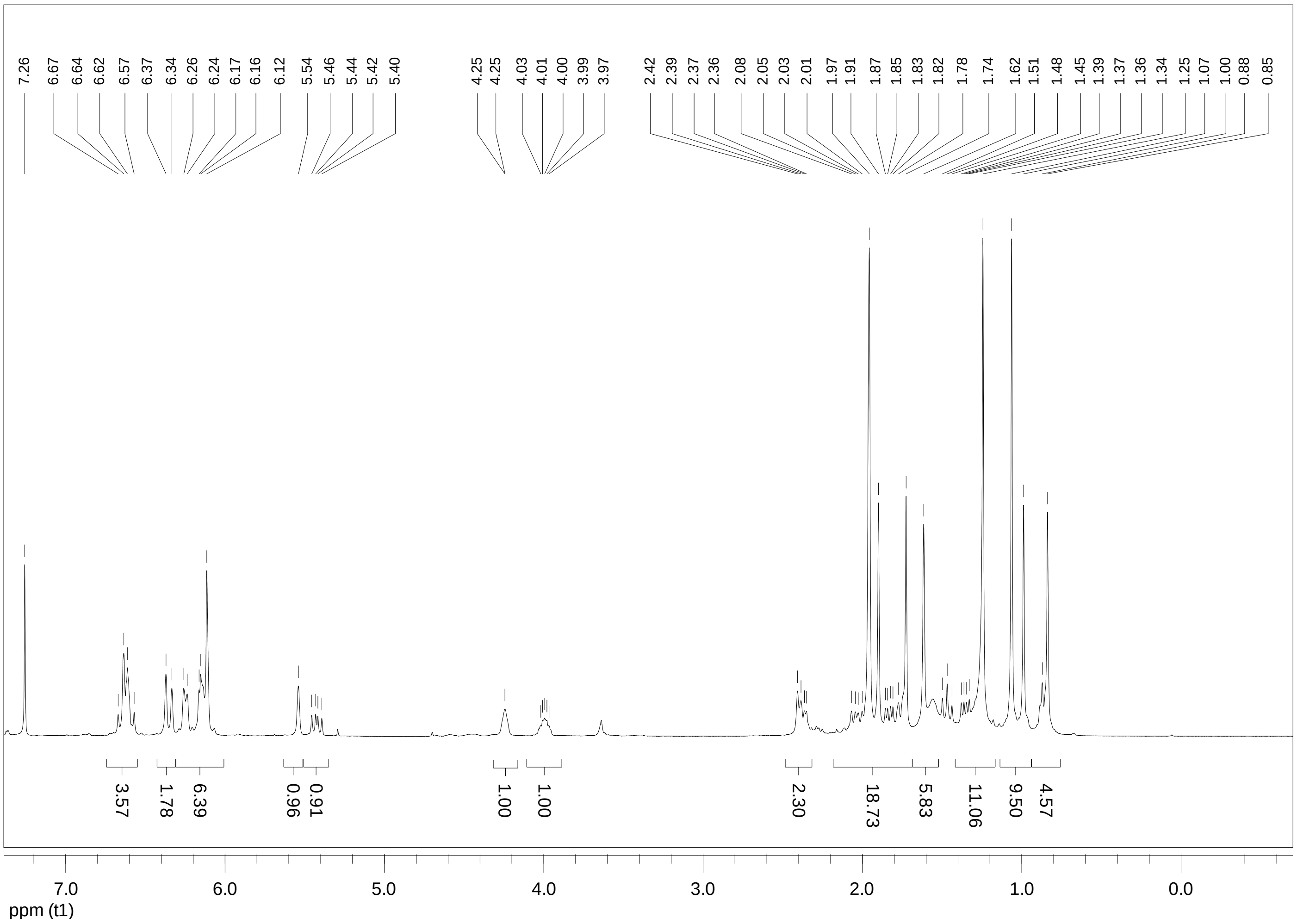

^{1}H-NMR (500 MHz, $CDCl_3$) δ 6.69 - 6.56 (4H, br m, H-11, H-15, H-11′, H-15′), 6.35 (2H, d, J = 14.8 Hz, H-12, H-12′), 6.28 - 6.22 (2H, br m, H-14, H-14′), 6.18 - 6.13 (3H, br m, H-14, H-8′, H-14′), 6.12 (2H, m, H-7, H-8), 5.54 (1H, s, H-4′), 5.43 (1H, dd, J = 15.3, 9.8 Hz, H-7′), 4.25 (1H, s, H-3′), 4.05 - 3.95 (1H, br m, H-3), 2.44 - 2.34 (2H, br m, H-4, H-6′), 2.04 (1H, dd, J = 17.0, 9.6 Hz, H-4), 1.97 (12H, s, H-19, H-20, H-19′, H-20′), 1.84 (1H, dd, J = 13.1, 5.8 Hz, H-2), 1.83 – 1.76 (1H, br m, H-2), 1.74 (3H, s, H-18), 1.62 (3H, s, H-18′), 1.48 (1H, t, J = 11.9 Hz, H-2), 1.36 (1H, dd, J = 13.1, 6.8 Hz, H-2′), 1.07 (6H, s, H-16, H-17), 0.85 (6H, s, H-16′, H-17′).

Figure S5. ^{1}H-NMR spectrum of the isolated (all-*E*)-lutein 5,6-epoxide

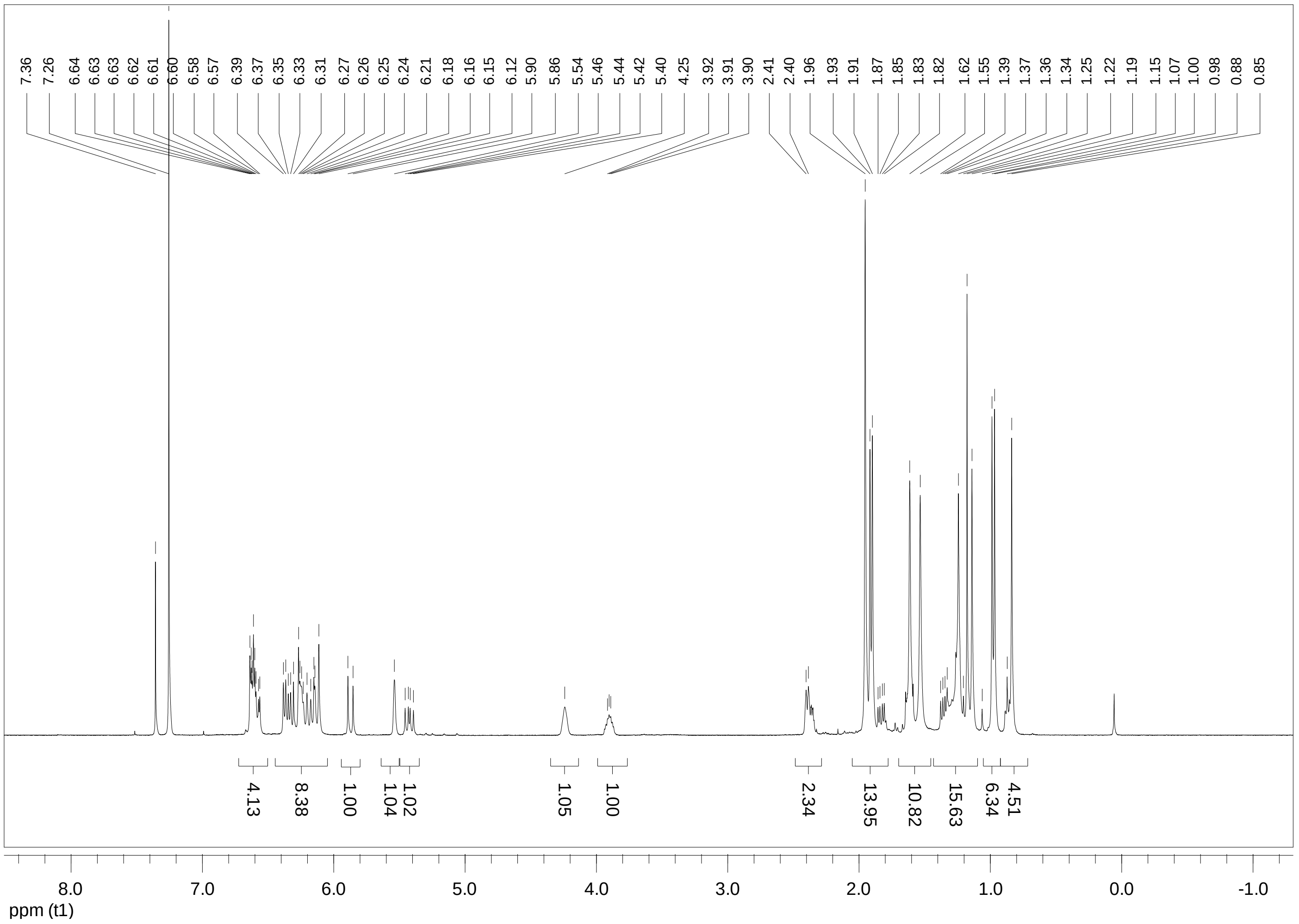

^{1}H-NMR (500 MHz, $CDCl_3$) δ 6.66 – 6.55 (4H, br m, H-11, H-15, H-11′, H-15′), 6.37 (1H, d, J = 14.9 Hz, H-12), 6.35 (1H, d, J = 14.9 Hz, H-12′), 6.29 – 6.22 (3H, br m, H-8, H-14, H-14′), 6.20 (2H, d, J = 15.5 Hz, H-10, H-10′), 6.17 – 6.09 (1H, br m, H-8′), 5.88 (1H, d, J = 15.5 Hz, H-7), 5.55 (1H, s, H-4′), 5.43 (1H, dd, J = 15.4, 9.8 Hz, H-7′), 4.25 (1H, s, H-3′), 3.96 – 3.86 (1H, br m, H-3), 2.43 – 2.34 (2H, br m, H-4, H-6′), 1.96 (6H, s, H-20, H-20′), 1.84 (1H, dd, J = 13.2, 5.9 Hz, H-2′), 1.83 (3H, s, H-19), 1.82 (3H, s, H-19′), 1.66 – 1.59 (5H, br m, H-2, H-4, H-18′), 1.36 (1H, dd, J = 13.2, 6.8 Hz, H-2′), 1.31 – 1.21 (1H, br m, H-2), 1.19 (3H, s H-18), 1.15 (3H, s, H-17), 1.00 (3H, s, H-17′), 0.98 (3H, s, H-16), 0.85 (3H, s, H-16′).

Figure S6. ^{1}H-NMR spectrum of the isolated (all-*E*)-violaxanthin

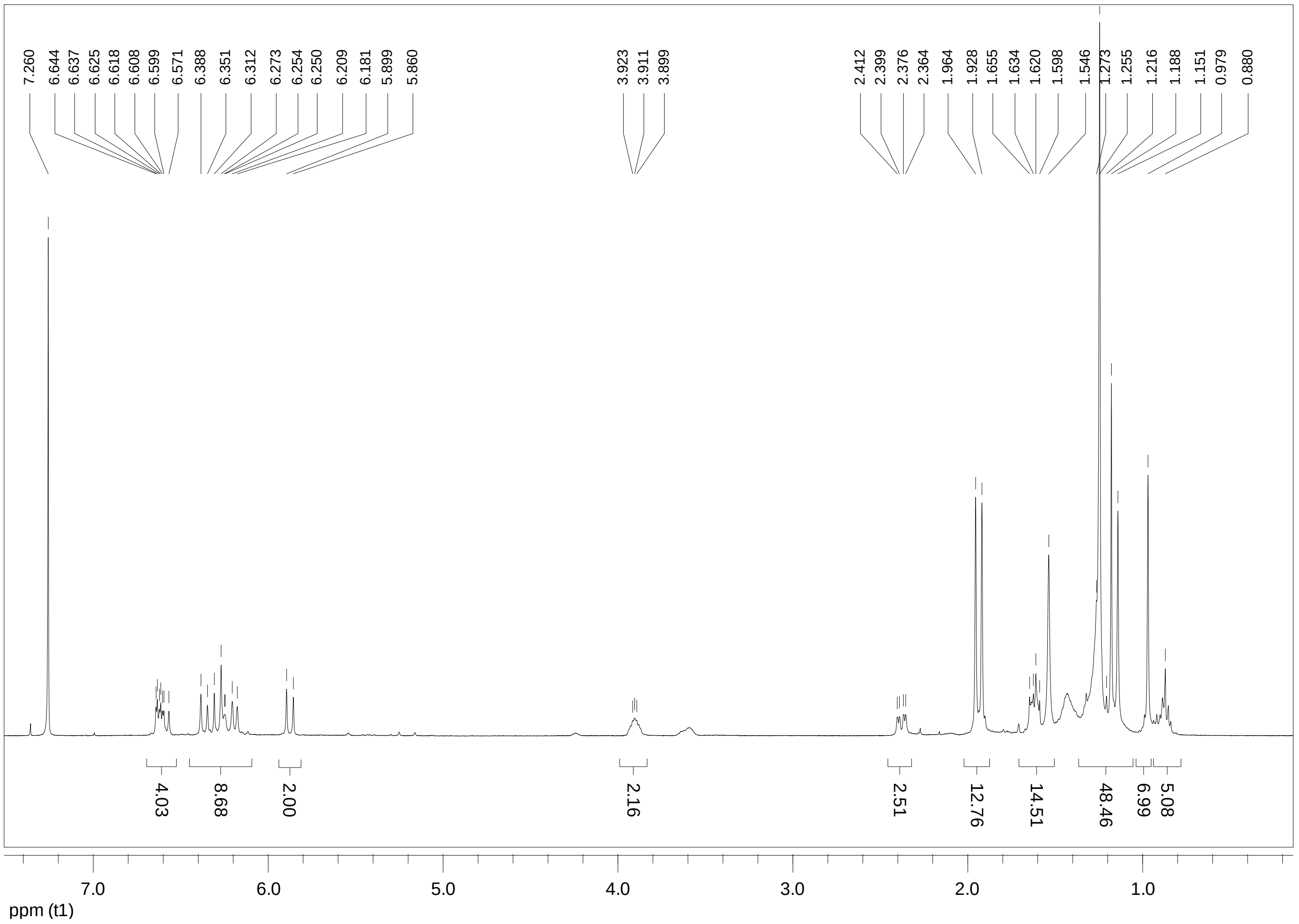

^{1}H-NMR (500 MHz, $CDCl_3$) δ 6.65 – 6.62 (2H, m, H-15, H-15′), 6.61 (2H, dd, J = 14.5, 10.7 Hz, H-11, H-11′), 6.37 (2H, d, J = 14.9 Hz, H-12, H-12′), 6.29 (2H, d, J = 15.5 Hz, H-8, H-8′), 6.25 (2H, m, H-14, H-14′), 6.20 (2H, d, J = 15.5 Hz, H-10, H-10′), 5.88 (2H, d, J = 15.5 Hz, H-7, H-7′), 3.95 – 3.86 (2H, br m, H-3, H-3′), 2.39 (2H, ddd, J = 14.5, 4.9, 1.3 Hz, H-4, H-4′), 1.96 (6H, s, H-20, H-20′), 1.93 (6H, s, H-19, H-19′), 1.63 – 1.57 (6H, br m, H-2, H-4, H-2′, H-4′), 1.19 (6H, s, H-18, H-18′), 1.15 (6H, s, H-17, H-17′), 0.98 (6H, s, H-16, H-16′).
